# The correlation between drug abuse belief, refusal of self-efficacy and drug craving of patients in the treatment of methadone replacement treatment

**DOI:** 10.3389/fpsyg.2026.1863585

**Published:** 2026-07-08

**Authors:** Jinhuo Cai, Hebai Dong, Tong-Yao Jin

**Affiliations:** 1 Wenzhou Seventh Peoples Hospital, Wenzhou, China; 2 Suan Sunandha Rajabhat University, Bangkok, Thailand

**Keywords:** addiction psychology, cognitive-behavioral mechanism, drug abuse beliefs, drug craving of patients, methadone maintenance treatment, refusal self-efficacy

## Abstract

This study investigates the intricate interrelationships among drug abuse beliefs (DAB), drug craving of patients (DCP), and refusal self-efficacy (RSE) in individuals undergoing methadone maintenance treatment (MMT). It establishes a theoretical foundation for optimizing psychosocial interventions and mitigating relapse risk among MMT patients. The study employed a purposive sampling method, administering questionnaires to patients receiving MMT at three methadone clinics in Wenzhou, utilizing localized measurement scales for DAB, DCP, and RSE. The analyses revealed three primary findings: (1) Erroneous DAB were significantly and positively correlated with elevated levels of DCP; (2) Rather than functioning solely in a sequential manner, both DAB and DCP acted as robust, independent, and parallel negative predictors of patients’ RSE; (3) The adverse effects of distorted cognitive beliefs on willpower were fundamentally intertwined with the immediate psychological experience of craving, collectively undermining behavioral confidence in maintaining abstinence. These conclusions enhance the understanding of the dual psychological vulnerabilities to relapse among Chinese MMT patients, underscoring the necessity of concurrently integrating cognitive belief restructuring and craving management into standard pharmacological treatments.

## 1 Introduction

In the context of global opioid dependence, substance use disorder (SUD) has increasingly been recognized as a chronic, relapsing condition that requires not only pharmacological stabilization but also sustained psychosocial and cognitive-behavioral intervention. Methadone maintenance treatment (MMT) remains one of the most widely implemented evidence-based interventions for opioid use disorder (D'Aunno et al., 2019; Ghanem et al., 2022), with well-documented effectiveness in reducing withdrawal symptoms, improving treatment retention, and decreasing illicit opioid use (Amato et al., 2013; Yahaya et al., 2026). However, recent clinical and theoretical advances have emphasized that pharmacological stabilization alone is insufficient to ensure sustained recovery (Fu et al., 2025; Toreyhi et al., 2025). Relapse is strongly influenced by patients’ cognitive, emotional, and motivational processes (Volkow and Blanco, 2023; Valencia and Peters, 2026). Accordingly, contemporary treatment guidelines increasingly highlight the importance of integrating psychosocial and cognitive interventions into MMT to enhance long-term behavioral regulation and relapse prevention (World Health Organization, 2009; Gkintoni et al., 2025).

A substantial body of literature has identified cognitive and motivational factors as central determinants of addiction outcomes. Among these, drug abuse beliefs (DAB) and refusal self-efficacy (RSE) have emerged as two particularly influential constructs (Cheesman and Read, 2023; Choi et al., 2013). DAB refers to maladaptive cognitive schemas and outcome expectations regarding drug use, such as the belief that drugs are effective for coping with stress or emotional distress (Essfioui, 2025). In contrast, RSE reflects an individual’s perceived capability to resist drug use in high-risk situations, grounded in Bandura’s social cognitive theory (Bandura, 1997). Empirical evidence consistently demonstrates that stronger RSE is associated with reduced relapse risk and improved treatment adherence, whereas maladaptive drug-related cognitions increase vulnerability to substance use behavior (Sampedro et al., 2026; Kadden and Litt, 2011; Barta et al., 2009). Nevertheless, existing research has predominantly examined these constructs in isolation, rather than within an integrated explanatory framework of addiction cognition.

DCP is widely recognized as one of the most proximal predictors of relapse among individuals receiving MMT. Craving is not only a physiological response to withdrawal but also a psychologically mediated state shaped by cognitive appraisal, emotional regulation, and contextual cues (Wilson, 2022; Gu et al., 2016; Fareed et al., 2010). Recent neurocognitive models conceptualize craving as a dynamic interaction between cognitive expectations and self-regulatory capacity, rather than merely a biological symptom (Moniz-Lewis et al., 2022). Empirical studies have shown that higher craving intensity is associated with increased relapse risk, poorer treatment adherence, and reduced long-term recovery outcomes (Vafaie and Kober, 2022; Higley et al., 2011). Although MMT can significantly reduce withdrawal-driven cravings, persistent psychological cravings remain a major challenge, even in stabilized patients. This suggests that pharmacological intervention alone does not fully address the cognitive-emotional mechanisms underlying the maintenance of cravings (Maddern et al., 2022; Chen et al., 2021; Shi et al., 2007).

From a theoretical perspective, Marlatt and Gordon (1985) cognitive-behavioral model of relapse provides an important foundation for understanding addiction processes (Marlatt and Gordon, 1985). This model posits that relapse is mediated by interactions between cognitive appraisals, coping responses, and self-efficacy beliefs (Lowe et al., 2008). Specifically, maladaptive outcome expectancies (e.g., DAB) may weaken coping confidence (RSE), thereby increasing vulnerability to craving and relapse in high-risk situations (Hunter-Reel et al., 2009). In this framework, self-efficacy serves as a central regulatory mechanism that translates cognitive beliefs into behavioral outcomes (Magill et al., 2025). However, the majority of empirical applications of this model have concentrated on alcohol or general substance use populations, with limited validation in populations receiving MMT, particularly within non-Western contexts, such as China.

In the Chinese context, MMT patients face additional structural and psychosocial challenges that may intensify cognitive vulnerability to craving. These include persistent social stigma, limited community reintegration support, and widespread misconceptions regarding methadone as a substitute addiction (Tang et al., 2024; Zou et al., 2015). Such contextual factors may reinforce maladaptive drug beliefs while simultaneously undermining patients’ perceived behavioral control, thereby creating a reinforcing cycle between cognition and craving. Although previous studies in China have confirmed that MMT reduces withdrawal symptoms and improves social functioning (Liu et al., 2026; Pan et al., 2015), systematic evidence regarding the interactive cognitive mechanisms linking DAB, RSE, and craving remains limited and fragmented.

A significant theoretical gap persists in the current literature: while existing studies acknowledge the relevance of DAB, RSE, and DCP, they have rarely investigated their joint predictive effects on craving intensity within a single analytical framework. Most prior research has examined these cognitive constructs in isolation. Consequently, the relative and sequential predictive weights of maladaptive beliefs and self-efficacy on actual craving experiences remain underexplored, particularly in culturally specific treatment contexts such as MMT in China.

Building on social cognitive theory, this study proposes that both DAB and RSE serve as critical cognitive factors that directly predict the intensity of DCP among MMT patients. Additionally, maladaptive DAB is anticipated to significantly negatively predict RSE. Therefore, this study aims to utilize multiple regression analysis to clarify the directional associations and predictive strengths among DAB, RSE, and DCP within a Chinese MMT population.

Therefore, this study aims to examine the interrelationships among DAB, RSE, and DCP among patients receiving MMT in China.

Based on the theoretical framework and prior empirical evidence, the following hypotheses are formulated:

*H1*: Drug abuse beliefs significantly and positively predict drug craving of patients.

*H2*: Drug abuse beliefs significantly and negatively predict refusal self-efficacy.

*H3*: Refusal self-efficacy significantly and negatively predicts drug craving of patients.

## 2 Materials and methods

### 2.1 Study design

This study employed purposive sampling to examine the associations between DAB, RSE, and DCP among individuals receiving MMT at three methadone clinics in Wenzhou. To ensure the representativeness of the sample and compliance with ethical standards, formal approval was obtained from the medical institution and relevant ethics authorities. Eligible patients were approached and informed about the study’s objectives and response procedures while waiting for their appointments. After obtaining signed informed consent, self-report questionnaires were distributed for completion.

#### 2.1.1 Inclusion criteria

To ensure that the target population accurately represented the research objectives, participants were required to meet the following inclusion criteria: (1) currently enrolled in a standardized MMT program; (2) aged 18 years or older; (3) meeting the diagnostic criteria for opioid use disorder (OUD) as defined by the DSM-5; (4) possessing sufficient reading and cognitive abilities to comprehend and independently complete the psychological self-report questionnaires; and (5) willing to participate in the study and provide written informed consent prior to data collection.

#### 2.1.2 Exclusion criteria

To minimize potential confounding factors and ensure data reliability, the following exclusion criteria were established: (1) individuals with severe cognitive impairment, intellectual disabilities, or diagnosed severe psychiatric disorders (e.g., schizophrenia or acute bipolar episodes) that could hinder accurate self-reporting; (2) individuals currently experiencing severe acute withdrawal symptoms or acute intoxication during the assessment period, as these conditions could significantly distort cognitive and craving evaluations; and (3) individuals who refuse to participate, withdraw consent during the process, or provide highly incomplete questionnaire responses (e.g., missing more than 10% of the core scale items).

### 2.2 Participants in the study

According to regulatory data from Wenzhou City, as of December 2024, approximately 300 drug users were receiving methadone substitution therapy across 11 counties and districts. This study surveyed patients aged 18 to 60 from three MMT clinics in Wenzhou. Out of 140 distributed questionnaires, 121 valid responses were obtained, resulting in a validity rate of 86.4%. Among the respondents, 78.51% were male and 21.49% were female. The educational levels of participants were predominantly junior high (49.59%) and primary school (26.45%). Most participants were married (58.68%), and 38.84% were unemployed. In terms of substance use, 82.64% reported smoking, 60.33% reported alcohol consumption, and 4.13% reported chewing betel nuts. The age of first drug use was predominantly between 18 and 25 years (41.32%). Most participants had attempted to quit drugs two to ten times (60.33%) and had a history of drug use spanning over 5 years (66.94%). Additionally, 25.62% reported having a criminal record. The methods of drug use were primarily intravenous injection (71 cases) and inhalation (89 cases), indicating their prevalence within the sample.

### 2.3 Research tools

This study employed a structured questionnaire as the primary data collection instrument. The questionnaire encompassed four key dimensions: demographic characteristics, DAB, DCP, and RSE. The sources and content of each scale are detailed below:

#### 2.3.1 Drug abuse beliefs scale

The standardized version of the DAB Scale (DABS), developed by Lin and Huang (2004), was utilized in this study. This scale comprises 26 items categorized into three dimensions: effect expectancy, self-devaluation and dependence, and underestimation of addiction. The scoring range for the scale is from 26 to 104, with higher scores reflecting a greater propensity to overestimate drug efficacy, experience more intense drug cravings, and exhibit a dismissive attitude toward the risks of addiction.

#### 2.3.2 Drug craving of patients scale

The standardized 10-item DCP Scale (DCPS), developed by Beck et al. (1993), was utilized in this study. Scores on the scale range from 10 to 40, with higher scores indicating a greater desire for drugs, an increased tendency to overestimate drug efficacy, a stronger compulsion to use drugs, and a more negligent attitude toward the risks associated with addiction.

#### 2.3.3 Refusal of self-efficacy scale

Drug RSE was assessed using the drug RSE Scale (RSES) for Substance Abusers, developed by Geng and Han (2008). This scale was adapted from international RSE instruments and refined through qualitative interviews and open-ended questionnaires to ensure cultural relevance within the Chinese context. The questionnaire comprises 17 items conceptualized across three dimensions: Environmental Factors, Negative Emotions, and Positive Emotions. Scores on the scale range from 17 to 85, with higher scores indicating a stronger ability to resist drug temptation, greater confidence in refusing drug opportunities, and improved maintenance of abstinence. Confirmatory factor analysis (CFA) of the original scale demonstrated good model fit (*χ*^2^/df = 1.61, RMSEA = 0.061, CFI = 0.97, NFI = 0.92, NNFI = 0.96), and internal consistency was satisfactory (total *α* = 0.921; subscales α > 0.823).

### 2.4 Data analysis

This study employed SPSS 26.0 statistical software for data analysis to explore the correlations among DAB, RSE, and DCP in patients undergoing methadone substitution therapy. Initially, descriptive statistics were utilized to analyze the basic demographic characteristics of the sample, with means and standard deviations calculated for each key variable to elucidate the overall trends of the subjects concerning various psychological constructs. Subsequently, Pearson Product–Moment Correlation analysis was conducted to investigate the linear relationships among the three variables. This analytical method is widely recognized in psychology and behavioral science research for assessing the degree of linear correlation between continuous variables and demonstrates robust reliability and validity. Following the correlation analysis, a hierarchical multiple regression was conducted to examine the primary predictive effects and to evaluate the incremental variance (△*R*^2^) explained by the focal constructs. To adequately control for potential confounding variables, demographic characteristics—including gender, education, and substance use history—were included as covariates in the first block of the regression model. Subsequently, the primary independent variables were sequentially entered into the model to explicitly assess their independent contributions to the dependent variable.

## 3 Results

### 3.1 Descriptive statistics

To understand the distribution and response trends of each latent construct within the sample, a descriptive statistical analysis of the main variables was conducted. The results indicated that the mean score for DAB was 2.37 (SD = 0.73), reflecting a low level of misconceptions about drug abuse among participants and suggesting a certain level of awareness regarding the harms of drugs. The sample distribution was relatively concentrated, with most participants exhibiting consistent attitudes toward drug abuse. The mean score for DCP was 2.67 (SD = 0.88), indicating that participants held some beliefs and perceptions about DCP. Furthermore, the mean score for RSE was 2.74 (SD = 1.12), suggesting that although participants recognized the harms of drug use, they still experienced uncontrollable cravings. Notably, there was significant variability in RSE, indicating that while some participants were confident in their ability to abstain from drugs, others lacked this confidence. Overall, all three constructs were at midpoint levels, Detailed data are presented in Table 1.

**Table 1:** Descriptive statistics and correlations among key constructs.

Variables	Maximum	Mean	SD	1	2	3
1. Drug abuse beliefs	4	2.37	0.73	1		
2. Drug craving of patients	4	2.67	0.88	0.749**	1	
3. Refusal of self-efficacy	5	2.74	1.12	−0.517**	−0.514**	1

**Correlation is significant at the 0.01 level (2-tailed).

### 3.2 Indicator reliability

According to the SPSS 26.0 analysis results presented in Table 2, the three higher-level latent constructs examined in this study—namely, DAB, DCP, and RSE—demonstrated strong reliability. The detailed analysis is as follows:

1. DAB: The CITC index for each dimension ranged from 0.462 to 0.732, while the Cronbach’s Alpha for the three sub-facets varied from 0.806 to 0.906, indicating that the reliability of the DAB scale items was relatively good. Although a few items exhibited slightly weaker reliability, their scores were not low enough to warrant elimination.
2. DCP: The CITC index for each item ranged from 0.644 to 0.797, and the Cronbach’s Alpha value was 0.935. This high reliability and strong internal consistency suggest that the constructs measured by these items are relatively clear.
3. Refusal of self-efficacy (RSE): The CITC index for each item ranged from 0.691 to 0.890, with a Cronbach’s Alpha value of 0.974, indicating that the items within the self-efficacy scale were very stable and demonstrated excellent internal consistency.

**Table 2:** Measurement index reliability test.

Drug abuse beliefs	CITC	Drug craving of patients	CITC	Refusal of self-efficacy	CITC
SDATC1	0.595	DCP1	0.793	RSE1	0.749
SDATC2	0.730	DCP2	0.731	RSE2	0.797
SDATC3	0.665	DCP3	0.783	RSE3	0.706
SDATC4	0.766	DCP4	0.748	RSE4	0.842
SDATC5	0.669	DCP5	0.675	RSE5	0.872
SDATC6	0.726	DCP6	0.644	RSE6	0.815
SDATC7	0.637	DCP7	0.731	RSE7	0.841
SDATC8	0.732	DCP8	0.747	RSE8	0.831
UDATS1	0.462	DCP9	0.797	RSE9	0.801
UDATS2	0.560	DCP10	0.776	RSE10	0.859
UDATS3	0.558			RSE11	0.890
UDATS4	0.792			RSE12	0.886
UDATS5	0.610			RSE13	0.836
UDATS6	0.693			RSE14	0.691
UDATS7	0.621			RSE15	0.835
TEES1	0.617			RSE16	0.859
TEES2	0.702				
TEES3	0.751				
TEES4	0.632				
TEES5	0.604				
TEES6	0.689				
TEES7	0.550				
TEES8	0.593				
TEES9	0.703				
TEES10	0.689				
TEES11	0.587				

### 3.3 Correlation analysis

The results of the correlation analysis are presented in Table 1. Overall, DAB and their subfacets exhibited significant correlations with both DCP and RSE (*p* < 0.01). The DAB, demonstrated a significant positive correlation with DCP (*r* = 0.749, *p* < 0.01) and a significant negative correlation with RSE (*r* = −0.517, *p* < 0.01). This indicates that stronger DAB are associated with increased DCP and decreased RSE. Furthermore, drug craving (DCP) exhibited a significant negative correlation with RSE (*r* = −0.514, *p* < 0.01), indicating that elevated levels of craving directly diminish patients’ confidence in their ability to maintain abstinence. These notable intercorrelations are consistent with theoretical expectations and establish a solid foundation for the ensuing hierarchical regression and mediation analyses.

### 3.4 Regression analysis

The results of the linear regression analysis are presented in Table 3. Initially, DAB exhibited a significant positive predictive effect on DCP (*B* = 0.893, SE = 0.072, *β* = 0.749, *t* = 12.346, *p* < 0.001, *R*^2^ = 0.562), indicating that for every one-unit increase in DAB, an individual’s DCP increased by approximately 0.89 units. DAB accounted for approximately 56.2% of the variance in DCP, suggesting that individuals with stronger DAB experience significantly heightened DCP.

**Table 3:** Results of regression analysis.

Path	*B*	SE	*β*	*t*	*p*	VIF	*R* ^2^
Drug abuse beliefs → Drug craving of patients	0.893	0.072	0.749	12.346	<0.001	1.000	0.562
Drug abuse beliefs → Refusal of self-efficacy	−0.789	0.120	−0.517	−6.590	<0.001	1.000	0.267
Drug craving of patients → Refusal of self-efficacy	−0.649	0.101	−0.514	−6.543	<0.001	1.000	0.265

Secondly, DAB significantly and negatively predicted RSE (*B* = −0.789, SE = 0.120, *β* = −0.517, *t* = −6.590, *p* < 0.001, *R*^2^ = 0.267), indicating that individuals with heightened DAB exhibited significantly lower RSE, explaining approximately 26.6% of the variance.

Furthermore, DCP also had a significant negative impact on RSE (*B* = −0.649, SE = 0.101, *β* = −0.514, *t* = −6.543, *p* < 0.001, *R*^2^ = 0.265), indicating that higher DCP was associated with lower RSE. The VIF values for all three models were 1.0, indicating no multicollinearity.

In summary, DAB not only directly enhance an individual’s DCP but also diminish their self-efficacy in resisting drug use. This finding supports the drug-related belief theory, which posits that an individual’s beliefs about drugs can significantly influence their behavioral tendencies and levels of self-efficacy.

### 3.5 Hierarchical regression analysis

This study employed hierarchical multiple regression analysis to examine the predictive effects of DAB and DCP on RSE among patients undergoing MMT; the results of the analysis are presented in Table 4.

**Table 4:** Hierarchical regression analysis predicting refusal self-efficacy.

	Predictors	Model 1 (covariates)		Model 2 (+DAB)		Model 3 (+DCP)	
		*β*	*t*	*β*	*t*	*β*	*t*
Control variables	Gender	0.111	1.235	0.103	1.283	0.107	1.349
	Education	0.079	0.870	−0.003	−0.033	0.006	0.073
	Marital status	−0.059	−0.650	−0.078	−0.954	−0.054	−0.661
	Occupation	0.153	1.649	0.101	1.202	0.059	0.701
	Smoking status	0.104	1.147	0.125	1.532	0.129	1.601
	Alcohol use	−0.262**	−2.926	−0.179*	−2.180	−0.155	−1.909
	Betel nut use	−0.047	−0.517	−0.014	−0.170	0.012	0.148
	Age at first drug use	−0.080	−0.835	−0.038	−0.442	−0.087	−0.995
	Number of detoxifications	−0.108	−1.051	−0.033	−0.352	−0.040	−0.436
	Years of drug use	0.093	0.948	0.088	0.993	0.072	0.827
	Criminal record	−0.132	−1.482	−0.055	−0.676	−0.028	−0.345
Block 2	DAB			−0.450***	−5.211	−0.254*	−2.050
Block 3	DCP					−0.273*	−2.161
Model fit	*R* ^2^	0.182*		0.346***		0.374*	
	Adjusted *R*^2^	0.099		0.274		0.298	
	*F*	2.203*		4.768***		4.910*	
	△*R*^2^	0.182		0.164		0.027	
	△*F*	2.203		27.157		4.670	

*N* = 121. Standardized coefficients (*β*) are reported. Tolerance = 0.69–0.94, VIF = 1.07–2.72, indicating no serious multicollinearity. *p* < 0.10, *p < 0.05, **p < 0.01, ***p < 0.001. DAB, drug abuse beliefs; DCP, drug craving of patients; RSE, refusal self-efficacy.

In Model 1, control variables—including demographic characteristics and substance use history—were first incorporated into the regression equation. The results indicate that, collectively, the control variables accounted for 18.2% of the variance in RSE (*F* = 2.203, *p* < 0.05). Notably, ‘current drinking habits’ emerged as a significant negative baseline predictor (*β* = −0.262, *t* = −2.926, *p* < 0.01), suggesting that patients with drinking habits exhibit relatively lower self-efficacy regarding the refusal of illicit drugs.

In Model 2, the inclusion of the core independent variable— DAB —resulted in a highly significant increase in the model’s explanatory power (△*R*^2^ = 0.164, △*F* = 27.157, *p* < 0.001). After controlling for the influence of covariates, DAB demonstrated a strong, significant negative predictive effect on RES (*β* = −0.450, *t* = −5.211, *p* < 0.001). This indicates that the more deeply patients harbor erroneous beliefs regarding drug abuse, the weaker their confidence becomes in maintaining abstinence and refusing drugs.

In Model 3, the variable of DCP was further incorporated. The results indicated that the inclusion of DCP once again contributed significant incremental explanatory power to the model (△*R*^2^ = 0.027, △*F* = 4.670, *p* < 0.05). In the final full model, both DAB (*β* = −0.254, *p* < 0.05) and DCP (*β* = −0.273, *p* < 0.05) emerged as significant negative predictors of RES. Notably, when DCP was included in the final model, the standardized coefficient of DAB decreased from −0.450 to −0.254, yet remained statistically significant. This attenuation suggests an overlapping predictive variance between DAB and DCP. Specifically, while erroneous beliefs independently undermine RSE, a portion of this detrimental effect is closely intertwined with the patient’s level of craving. Nevertheless, the sustained significance confirms that both cognitive beliefs and physiological/psychological craving are robust, distinct parallel predictors of patients’ confidence in maintaining abstinence.

Furthermore, collinearity diagnostics indicate that the tolerance values for all predictor variables range from 0.69 to 0.94, while the Variance Inflation Factors (VIF) range from 1.07 to 2.72. These values fall well below the conventional threshold of 10, suggesting that the model is free from severe multicollinearity issues and that the analytical results are robust and reliable (Table 4).

## 4 Discussion

### 4.1 Interrelationships among drug abuse beliefs (DAB), drug craving of patients (DCP), and refusal self-efficacy (RSE) in MMT patients

The present study elucidates the complex interplay between DAB, DCP, and RSE among patients undergoing MMT. Our findings demonstrate a significant positive association between DAB and self-reported levels of DCP, suggesting that maladaptive cognitions regarding drug use are inextricably linked to the intensity of craving. This aligns with a robust body of literature indicating that craving is not merely a physiological consequence of withdrawal but is profoundly shaped by an individual’s cognitive expectations (Tiffany et al., 2012). Importantly, when patients hold the maladaptive belief that relapse is an inevitable outcome, this cognitive distortion significantly contributes to their actual relapse risk by consciously magnifying the subjective experience of craving (Minervini et al., 2011; Vafaie and Kober, 2022). Previous studies utilizing craving belief assessments, such as the Chinese Craving Beliefs Questionnaire (CCBQ), have similarly underscored the high prevalence of these cognitive distortions among heroin users and their critical role in fueling compulsive drug-related cognition (Chang et al., 2011). Rather than being a simple prerequisite for such cognitions, intense DCP operates as a persistent state of desire that interacts bidirectionally with underlying drug-related beliefs (Badger et al., 2007). Furthermore, although some previous evidence linking craving beliefs to diminished treatment motivation originated from populations such as Narcotics Anonymous, these mechanisms are highly translatable to the MMT context, where such cognitive distortions similarly increase the likelihood of substance use (Altınöz et al., 2016).

Beyond exacerbating cravings, these cognitive distortions fundamentally undermine a patient’s confidence in their ability to maintain abstinence. Consistent with Bandura (1999) social cognitive theory, which posits that beliefs about personal agency dictate behavioral outcomes, our results revealed that stronger DAB significantly and inversely predicted RSE. As patients’ maladaptive beliefs about drug use intensify, their perceived capacity to resist use logically deteriorates. Empirical evidence corroborates this dynamic, demonstrating that individuals burdened by pronounced DAB exhibit lower self-efficacy and diminished motivation to alter addictive behaviors (Moeini et al., 2022; Torrecillas et al., 2015). Consequently, this deficit in RSE leaves patients less equipped to navigate craving-inducing triggers, a vulnerability consistently linked to elevated relapse rates across addiction literature (Chen et al., 2021; Kadden and Litt, 2011; Taylor and Williams-Salisbury, 2015).

The immediate psychological burden of craving acts as a distinct mechanism that undermines self-efficacy. Our analysis confirmed a significant negative association between DCP and RSE, thereby substantiating the classic ‘cognitive model of drug craving’ (Tiffany, 1990). According to this model, acute experiences of craving disrupt self-regulation and foster a sense of futility; when individuals anticipate that resisting cravings will be exceedingly difficult, their confidence in their behavioral capabilities diminishes (Hayaki et al., 2021). The detrimental impact of craving on self-efficacy is well-documented (Mason et al., 2009), with recent studies consistently demonstrating that increased craving tendencies correlate with significant deficits in self-efficacy (Khazaee-Pool et al., 2025; Sheykhnezhad and Seyedfatemi, 2019). Ultimately, our findings suggest that DAB and DCP work synergistically to erode RSE. When patients experience intense cravings driven by maladaptive beliefs, they systematically underestimate their capacity to abstain, creating a precarious psychological environment that elevates the risk of relapse.

### 4.2 Dual mechanisms: the sequential and parallel impact of drug abuse beliefs (DAB) and drug craving of patients (DCP) on refusal self-efficacy (RSE)

The initial findings support a sequential cognitive-emotional mechanism, demonstrating that DAB directly exacerbates DCP, which subsequently decreases RSE. However, further hierarchical regression analysis revealed a more sophisticated parallel mechanism. Notably, when DCP was introduced into the final regression model, the standardized coefficient of DAB significantly decreased from −0.450 to −0.254, while retaining its statistical significance. This statistical attenuation does not imply simple linear mediation; rather, it underscores a profound overlap in predictive variance. It illustrates that a patient’s cognitive distortions (DAB) and immediate cravings (DCP) are fundamentally interconnected risk factors. While DAB exacerbates cravings, both serve as independent, parallel predictors that jointly undermine a patient’s self-efficacy. This dual mechanism—operating both sequentially and in parallel—provides insight into why some patients struggle to fully overcome dependence even after undergoing MMT.

### 4.3 Clinical and practical implications

The intertwined nature of DAB and DCP has significant implications for clinical practice. Current rehabilitation systems, particularly within the Chinese sociocultural context—characterized by intense addiction stigma and fragmented community support (Wu et al., 2013; Zou et al., 2015)—tend to rely predominantly on methadone pharmacotherapy. While MMT effectively manages baseline physical withdrawal symptoms, it addresses only the physiological component of the dual vulnerability outlined in our model. Cognitive distortions independently undermine self-efficacy and simultaneously exacerbate cravings, rendering purely pharmacological interventions inadequate. When patients harbor misconceptions—such as perceiving methadone as merely ‘another addictive substance’ or believing that relapse is unavoidable—these negative beliefs significantly diminish treatment efficacy. To optimize treatment outcomes, clinical programs must move beyond isolated approaches. Localized interventions should comprehensively integrate psychoeducation and cognitive behavioral therapy (CBT) to systematically restructure maladaptive drug beliefs. Additionally, programs must incorporate self-efficacy enhancement training, including coping skill rehearsal and emotional regulation, to protect patients against episodic spikes in cravings.

## 5 Limitations and future research directions

While this study addresses critical gaps in the existing literature, several limitations present opportunities for future research. First, the cross-sectional design limits the ability to establish definitive causal trajectories among DAB, DCP, and RSE. Although the findings are consistent with the cognitive-emotional-behavioral chain central to addiction theory, future research should utilize longitudinal designs to capture the dynamic developmental trajectories of these interrelated variables across different treatment stages. Second, the reliance on self-reported data from MMT outpatients may introduce social desirability bias. Future studies should consider incorporating objective physiological or behavioral measures of craving. Lastly, to gain a deeper understanding of the mechanisms underlying DAB, future research should investigate the broader social, psychological, and biological determinants of DAB. Cross-cultural studies and qualitative action research across diverse patient populations are strongly recommended to validate and expand upon the dual-vulnerability model identified in this study.

## 6 Conclusion

This study systematically investigates the complex psychological mechanisms influencing the outcomes of MMT, moving beyond traditional linear perspectives to reveal a ‘dual-vulnerability’ mechanism underlying relapse risk. Specifically, erroneous DAB act sequentially as catalysts that intensify patients’ DCP, while both factors simultaneously serve as robust, independent, and parallel negative predictors of RSE. The detrimental impact of deeply ingrained cognitive distortions on willpower is fundamentally intertwined with the immediate psychological experience of craving, which synergistically undermines a patient’s behavioral confidence in maintaining abstinence. Consequently, this intertwined dual mechanism highlights the inadequacy of relying solely on the pharmacological stabilization provided by MMT. To achieve optimal rehabilitation outcomes, clinical programs must implement integrated psychosocial interventions that concurrently combine cognitive belief restructuring to correct maladaptive DAB with coping-skill enhancement training to manage episodic DCP, thereby effectively fortifying patients’ RSE and preventing relapse.

## Data Availability

The original contributions presented in the study are included in the article/supplementary material, further inquiries can be directed to the corresponding author.
